# The sintering kinetics of shellfish porcelain reinforced by sepiolite nanofibres

**DOI:** 10.1098/rsos.180483

**Published:** 2018-10-17

**Authors:** Li Tian, Lijuan Wang, Kailei Wang, Yuedan Zhang, Jinsheng Liang, Yi Zhang

**Affiliations:** 1 Key Laboratory of Special Functional Materials for Ecological Environment and Information (Hebei University of Technology), Ministry of Education, Tianjin 300130, People's Republic of China; 2 Institute of Power Source and Ecomaterials Science, Hebei University of Technology, Tianjin 300130, People's Republic of China; 3 Hunan Key Laboratory of Mineral Materials and Application, Central South University, Changsha 410083, People's Republic of China

**Keywords:** sepiolite, nanofibre, shellfish porcelain, reinforce, sintering kinetics

## Abstract

The work investigated the effect of sepiolite nanofibres on mechanical properties and sintering behaviour of shellfish porcelain. Samples of shellfish porcelain reinforced by sepiolite nanofibres were fired in an electric furnace at 1150, 1200 and 1250°C for a period of 80, 100, 120 and 140 min. Sintered samples were characterized by flexural strength, fracture toughness, scanning electron microscopy, transmission electron microscopy and X-ray diffraction. The results showed that 2 wt% sepiolite nanofibres could increase the flexural strength and fracture toughness of the porcelain bodies through the fibre pullout and the weak interface mechanisms. Sintering activation energies were determined according to the linear shrinkage results. It is found that the liquid-phase sintering mechanism of shellfish porcelain with sepiolite nanofibres is a diffusion mechanism. Porcelain without sepiolite is controlled by volume diffusion, and eventually, the grain boundary diffusion began to appear with the increase of sepiolite addition.

## 1. Introduction

Bone china is a kind of unique ceramic in terms of appearance, exceptionally white and translucent, which makes it the most expensive tableware around the world [1]. The typical composition of a modern commercial bone china is 25 wt% clay, 25 wt% fluxing material and 50 wt% bone ash [2]. Traditional bone china uses animal bone ash as raw material, and now synthetic bone ash has emerged, whose performance is not much different from that of traditional bone ash [3]. Based on the inspiration of bone china, the application of seashell in ceramic materials provides a new idea for the development of bone china. It is a kind of bone china, known as shellfish porcelain, which possesses all the merits of bone china. However, the brittleness and small toughness limit the application in some special fields [4]. Such non-negligible properties motivate research concerning how to increase the toughness as one of the most attractive topics.

Traditionally, several methods have been used to toughen the shellfish porcelain. Particulate-, whisker- and fibre-reinforced ceramic composites are common ways to lead to crack bridging and deflection upon crack propagation [5,6]. The most commonly used method is to add nanofibres to reinforce the shellfish porcelain [7]. The experiments of several authors [8,9] show that the interface, an important part formed in composite materials, is related to the mechanical properties of composites and the environmental performance. Because of the high cost and health hazard associated with the preparation of synthetic nanofibres, the application of natural nanofibre materials may provide the possibility to solve the problems mentioned above.

Sepiolite is a natural hydrated magnesium silicate clay mineral with a microfibrous morphology and good sorptive property. It belongs to the structural family of 2 : 1 phyllosilicates with (Si_12_Mg_8_O_30_)(OH)_4_(OH_2_)_4_·8H_2_O as the theoretical unit cell formula. It has nanoporous channels with dimensions of 0.37 nm × 1.06 nm, running parallel to the length of the fibres [10,11]. Owing to its particular structure with tunnels, it has the properties of adsorption, rheology and catalysis. And thus, it finds applications in a variety of industries including cosmetics, ceramics, detergents, paper and paint [12–18]. Sepiolite mostly appears in the form of nanofibre or fibre bundle when observed using transmission electron microscopy (TEM) and scanning electron microscopy (SEM), which gives it a good foundation to become a toughening material [19–21]. These could induce that certain amount of sepiolite dispersed in the ceramic matrix can improve the mechanical properties of shellfish porcelain. Especially, sepiolite is a kind of cheap and abundant mineral resource, which will decrease the cost of the toughening of shellfish porcelain. Ran *et al.* [22] have applied sepiolite to bone china and found that sepiolite is beneficial to improve its mechanical strength. However, its sintering kinetics and the phase reaction of the weak interface are not clear. Therefore, the above-mentioned problems need to be solved.

In this work, sepiolite mineral nanofibres processed in our laboratory were used to reinforce shellfish porcelain. An attempt was also made to research the densification behaviour of samples with varying amount of sepiolite addition at different sintering temperatures and sintering times by using linear shrinkage. The mechanical properties and sintering kinetics of reinforced shellfish porcelain were systematically investigated.

## 2. Material and methods

### 2.1. Materials

Shellfish porcelain was traditionally produced from the raw materials of shell powder, quartz, Fangzi soil etc. The chemical compositions of raw materials for producing shellfish porcelain are presented in table 1.

**Table 1.** The chemical compositions of raw materials for producing shellfish porcelain (wt%).

material	SiO_2_	Al_2_O_3_	Fe_2_O_3_	TiO_2_	CaO	MgO	K_2_O	Na_2_O	burning loss
shell powder	2.15	0.89	0.45	—	53.12	0.42	0.21	0.11	41.78
Datong soil	44.64	38.82	0.17	—	0.48	0.20	0.44	0.20	15.83
Fangzi soil	57.22	26.76	1.31	0.79	0.49	0.35	1.56	0.10	11.19
Yixian soil	69.69	11.90	0.34	0.03	5.87	1.64	0.10	1.53	8.54
potash feldspar	65.02	19.30	0.09	—	—	—	12.72	1.47	0.33
quartz	99.70	0.10	0.13	—	—	—	—	—	—
Xushui soil	69.39	20.48	0.98	0.15	0.70	0.15	2.68	0.13	5.46
Longyan soil	79.01	12.43	0.26	0.02	0.64	0.28	1.56	0.84	5.26

Raw sepiolite used in this study was obtained from Nanyang City, Henan Province, China, and its relative density was 2.4–2.6 g cm^−3^. It was ground by a miller and sieved with a 38 μm sieve. To remove most impurities in raw sepiolite, purification was carried out by the sedimentation method firstly and then crushed by air stream. The chemical composition of the sepiolite is shown in table 2, and the main chemical components were SiO_2_ and CaO. Finally, the solid was dried at 110°C for 2 h and ground to sepiolite nanofibres, as shown in figure 5*b*.

**Table 2.** Chemical composition of sepiolite.

oxide	SiO_2_	CaO	MgO	Al_2_O_3_	Fe_2_O_3_	MnO	TiO_2_	K_2_O	Na_2_O	total
content (wt%)	39.7	32.4	17.0	6.20	3.46	0.40	0.27	0.21	0.20	99.84

### 2.2. Preparation

**Figure 1.**
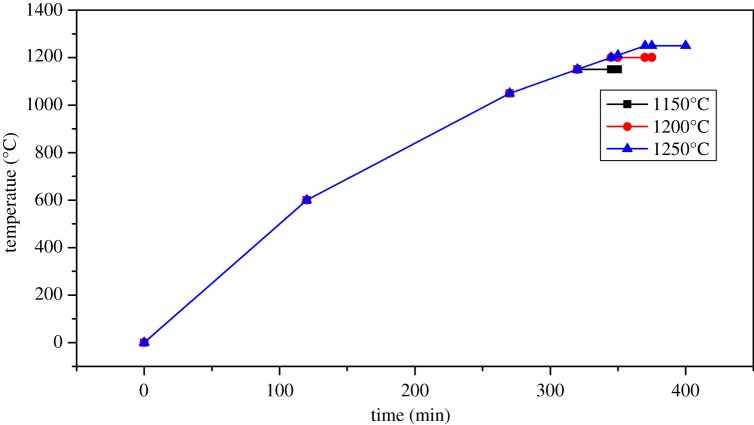
The sintering system curve.

**Figure 2.**
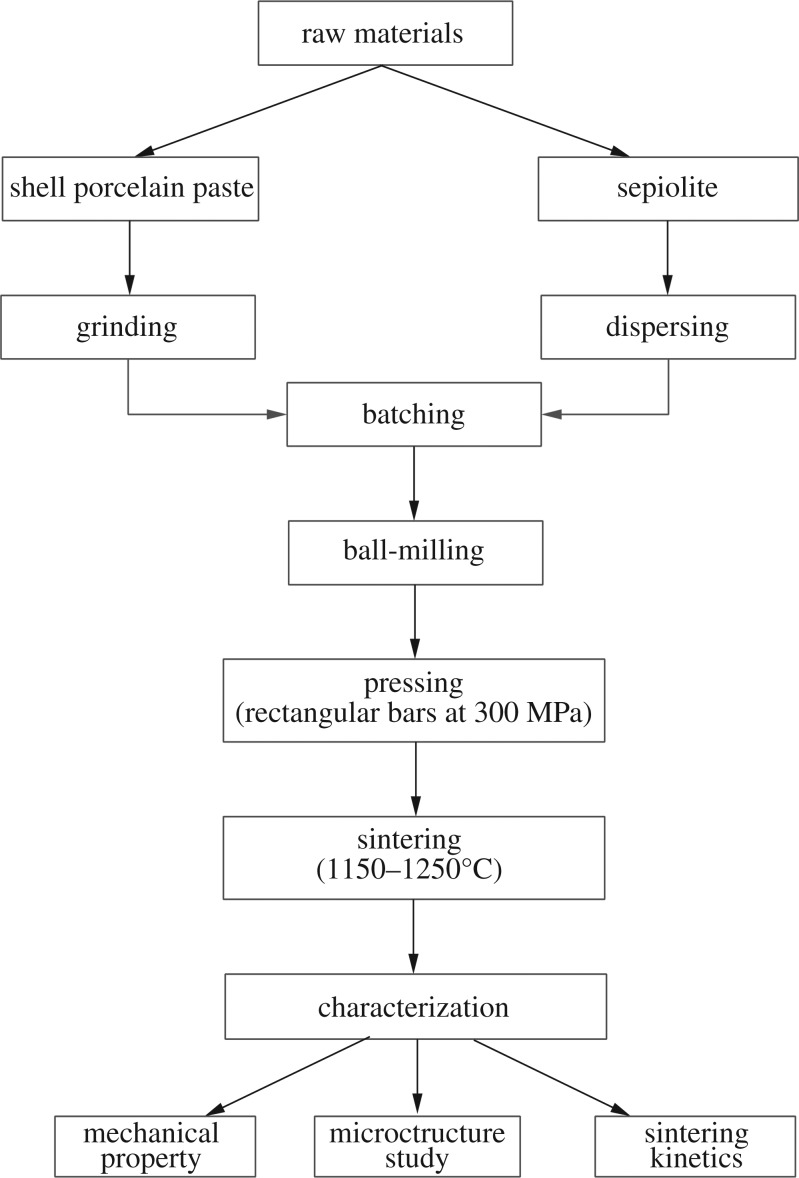
Flow chart for the processing and characterization of sepiolite nanofibres reinforced ceramic.

According to the original recipe in slurry, sepiolite nanofibres were added to it by mass of 0, 1, 2, 3 and 4 wt%. The body without sepiolite nanofibres was denoted by S0, and others with 1, 2, 3 and 4 wt% were denoted by S1, S2, S3 and S4, respectively. Green bodies with shell powder, china clay and other materials were milled to particle size and then intimately mixed. The prepared sepiolite nanofibres were added, respectively, to the slurry and milled for 30 min (the ball in the mill is corundum ball, its Moh's hardness is 9, the ratio of the ball and material in weight is 1 : 1 and the ball mill speed is 800 r.p.m.). After the powder was sieved by 200 mesh and deironing three times, samples of 40 mm × 6 mm × 4 mm were formed by dry pressing (the moulding pressure was 300 MPa and the holding time was 5 min), and every sample's weight was 2.5 g. Then, five samples marked as S0, S1, S2, S3 and S4 were studied in the laboratory. After shaping, samples were fired in an electric furnace with a heating rate in figure 1 at 1150, 1200 and 1250°C for a period of 80, 100, 120 and 140 min. Next, the fired samples produced by the steps of preparing the ceramic shown in figure 2 were cooled down to room temperature in the furnace.

### 2.3. Characterization techniques

The flexural strength was measured by the three-points bending method with a span of 20 mm and a cross-head speed of 0.5 mm min^–1^ on 40 mm × 6 mm × 4 mm test bars polished to an average surface roughness less than 0.1 mm with a microcomputer control electron universal tester (model 6104), based on CNS GB/T 6569-2006. The fracture toughness was determined on bars of the above size by a three-point single-edge notched beam method with a span of 20 mm and a cross-head speed of 0.5 mm min^–1^. The depth of notch groove was half of the height of test sample and the groove width was less than 0.28 mm. These are based on CNS GB/T 23806-2009. The microstructure was characterized with a scanning electron microscope (JSW-6700F, Holland) and a transmission electron microscope (JEM-2100, Japan), and the crystal structure was analysed by X-ray diffraction (XRD; XL30, Holland).

### 2.4. Sintering kinetics

The study of sintering kinetics and mechanisms to evaluate the effect of sepiolite nanofibres on sintering activation energy of sintered samples was based on linear shrinkage measurements, to measure the size of the ceramic body without sintering and to mark it as *L*_0_. After sintering, the length (*L*) of each sample which was prepared at different conditions was measured and the linear shrinkage rate expressed as Δ*L*/*L*_0_ (Δ*L* = *L*_0_ − *L*) is calculated. The early sintering kinetic equations proposed by Singh and co-workers [23,24] are used to study the sintering activation energy of shellfish ceramics toughened with sepiolite nanofibres and to determine the type of sintering mechanism:

2.1 $$\mathrm{lg}\frac{\Delta L}{L_{0}}=m\;\mathrm{lg}\;t+\mathrm{lg}k.$$

To calculate sintering activation energy, the Arrhenius equation is used:

2.2 $$k=\frac{1}{t}=A\exp\left(-\frac{Q}{RT}\right),$$

where △*L*/*L*_0_ is the relative reduction of the ceramic body with sepiolite nanofibres, *m* is reaction order, *t* is holding time, *k* is the sintering rate constant, *Q* is the sintering activation energy, *R* is the gas constant, *T* is absolute temperature, and *A* is a constant associated with interfacial tension, diffusion coefficient and particle radius.

## 3. Results and discussion

### 3.1. Flexural strength and fracture toughness of shellfish porcelain

**Figure 3.**
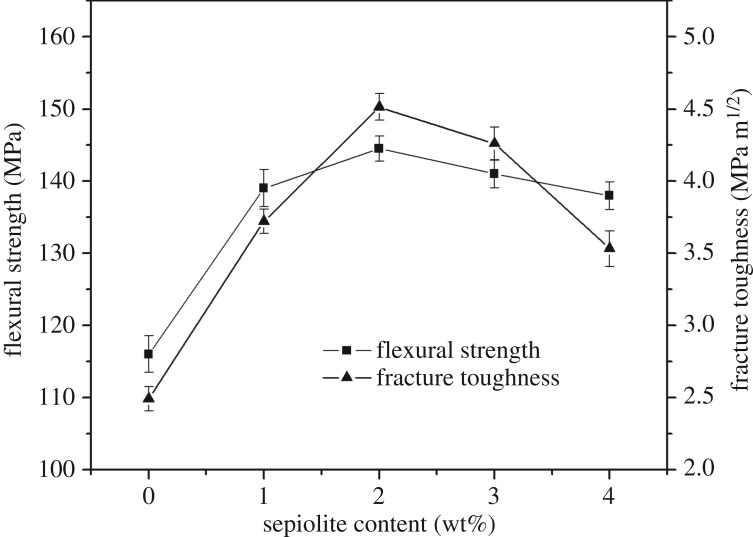
Effect of sepiolite nanofibres on the flexural strength and fracture toughness of shellfish porcelain.

The mechanical properties of shellfish porcelain can reach the maximum value when the amount of sepiolite nanofibres is 2 wt% and the sintering temperature is 1200°C. However, the shellfish porcelain without adding sepiolite nanofibres reaches the maximum value at 1250°C. Namely, the addition of sepiolite nanofibres plays a key role in reducing the sintering temperature. Figure 3 shows the flexural strength and fracture toughness as a function of sepiolite nanofibre addition at the sintering temperature of 1200°C.

Under the experimental conditions, the maximum flexural strength of shellfish porcelain has a value of 144.5 MPa when the quantity of 2 wt% sepiolite nanofibres are added, which is approximately 24.6% higher than that of shellfish porcelain without sepiolite nanofibres at 1200°C. And, the flexural strength decreases slowly with the increase of sepiolite nanofibre content. The fracture toughness of samples has the same tendency as that of the flexural strength. The sepiolite nanofibres can improve the fracture toughness of shellfish porcelain when their content is less than 2 wt% (figure 3). Particularly, the fracture toughness on average can reach 4.51 MPa m^1/2^ when the sepiolite nanofibre addition is 2 wt%. However, it suggests that the excessive addition will decrease the fracture toughness which is only 3.53 MPa m^1/2^ in the case of sepiolite nanofibre content of 4 wt%.

### 3.2. Microstructure and phase analysis

**Figure 4.**
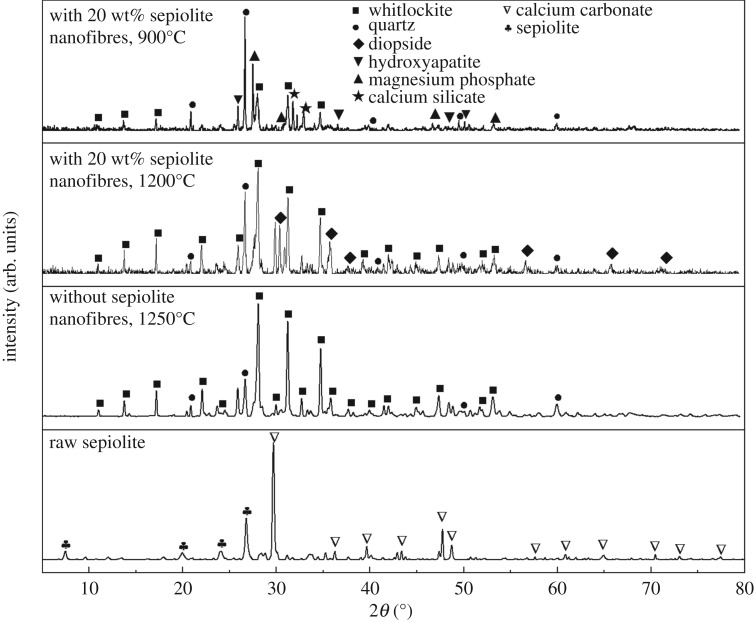
XRD patterns of sepiolite and ceramics with or without sepiolite nanofibres at different temperatures.

The XRD patterns of sepiolite and ceramics with or without sepiolite nanofibres at different temperatures are shown in figure 4. According to the raw materials we used, the sepiolite is contaminated with a small amount of calcium carbonate (CaCO_3_) impurities and the main crystalline phases of the ceramic without sepiolite nanofibres at 1250°C are whitlockite (Ca_3_(PO_4_)_2_) and quartz (SiO_2_). After adding the sepiolite nanofibres, the crystalline phases of the sample calcined at 1200°C become more complex than before because of diopside (CaMgSi_2_O_6_), indicating that the formation of diopside is closely related to the addition of sepiolite nanofibres. XRD analysis suggests that the sintered sample with 20 wt% sepiolite nanofibres at 900°C has the main crystalline phases such as whitlockite (Ca_3_(PO_4_)_2_), hydroxyapatite (Ca_5_(OH)(PO_4_)_3_) and quartz (SiO_2_). It is found that hydroxyapatite can remove the hydroxyl group to form the final whitlockite and the pore structure of sepiolite nanofibres becomes loose at elevated temperature. Magnesium phosphate and calcium silicate can enter into the pores of sepiolite nanofibres and react with quartz, indicating that the calcium silicate is the result of the reaction of lime produced by the decomposition of calcite contained in sepiolite and the quartz in the body. The reaction equation can be expressed as follows:

$$\begin{array}{rl} & \mathrm{CaC}O_{3}\to \mathrm{CaO}+CO_{2}↑\;\mathrm{CaO}+\mathrm{Si}O_{2}\to Ca_{2}\mathrm{Si}O_{4}\\ & 3Ca_{2}\mathrm{Si}O_{4}+Mg_{3}{\left(PO_{4}\right)}_{2}+3\mathrm{Si}O_{2}\to 3\mathrm{CaMgS}i_{2}O_{6}+Ca_{3}{\left(PO_{4}\right)}_{2}. \end{array}$$

**Figure 5.**
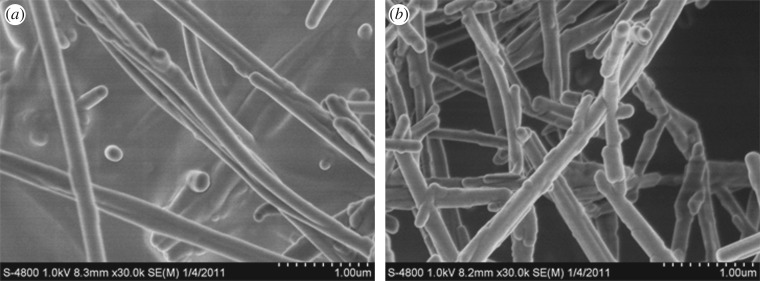
SEM images of section of sepiolite. (*a*) Raw sepiolite. (*b*) Jet mill grinding sepiolite.

Microstructural investigations carried out by SEM of raw sepiolite nanofibres and treated sepiolite by jet mill reveal various features as shown in figure 5. It can be noted that the raw sepiolite mineral fibres are adhered to each other by a kind of cementing material (figure 5*a*); however, the condition can be solved by using the method of physical airflow. Owing to the existence of residual cementing material, the treated fibres, shown in figure 5*b*, exist in a variety of single forms and their structure is loose, and the surface is rough.

**Figure 6.**
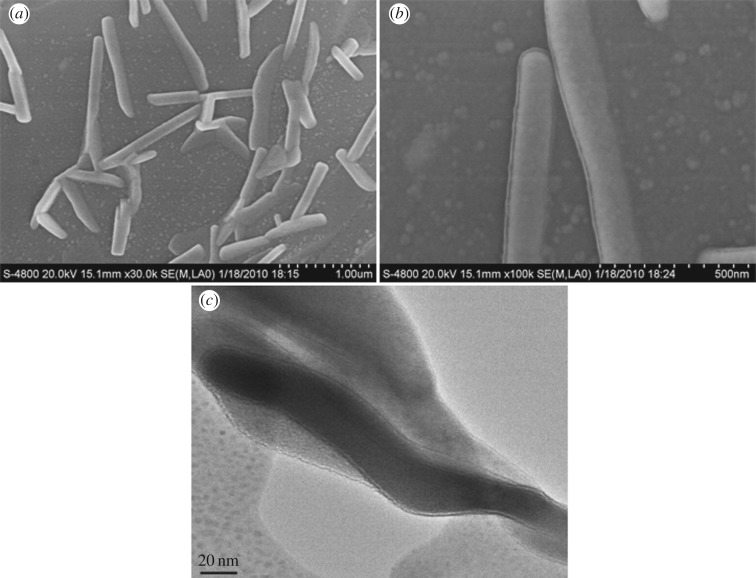
SEM photomicrographs (*a,b*) and TEM image (*c*) of sepiolite nanofibres in shellfish porcelain.

Some nanofibres were found in the shellfish porcelain with sepiolite nanofibres, as shown in figure 6. And, a new interface layer (figure 5*b*) is formed between the ceramic substrate and nanofibre. From figure 6*c*, there is a circle of narrow gap between the new interface layer and nanofibres and this narrow gap makes a weak combination between the fibre and the matrix. The reason for the formation of the weak interface is the reaction of sepiolite nanofibre and the hydroxyapatite in the matrix that happened in the process of ceramic sintering, as explained above. In the high temperature stage, the crystal structure of sepiolite mineral fibres is destroyed, an atomic structure rearrangement occurs and a new phase is formed. Along with the phase change process, on the one hand, the surface of sepiolite nanofibres reacts with the surrounding ceramic substrate, and on the other hand, the hydroxyapatite of ceramic matrix goes into the sepiolite ducts and reacts with the fibres. The channel structure collapse in the phase change process means that the fibres' surface breaks away from the substrate. The weak combination interface will cause plenty of micro-cracks and also supplemented by a contribution of the pullout of nanofibres when the porcelain is in service. When the pullout is accompanied by micro-cracks, additional strain energy must be supplied to hold back the crack. Moreover, energy consumption of fibre pullout also can hold back.

### 3.3. Sintering kinetics and mechanisms

**Figure 7.**
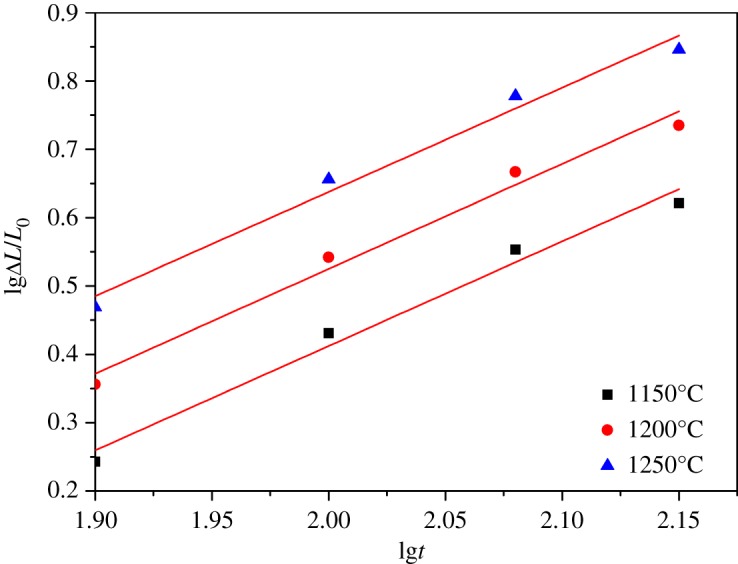
Relation of lgΔ*L*/*L*_0_ and lg*t* for S0 at different sintering temperature.

**Figure 8.**
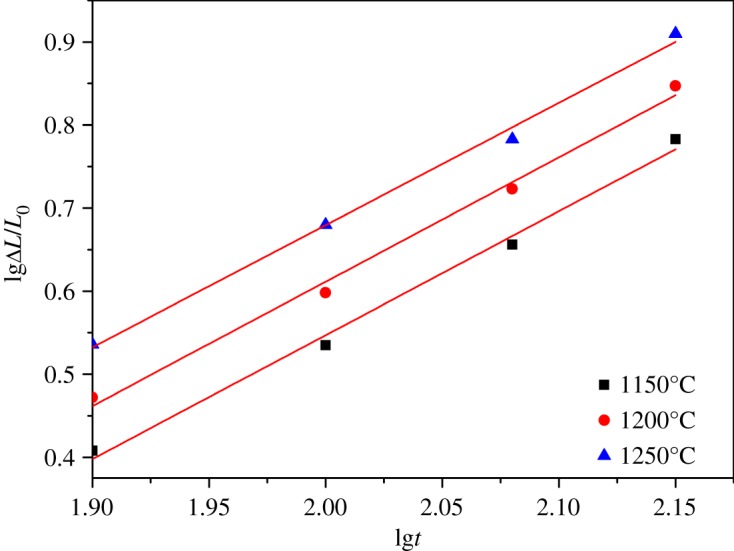
Relation of lgΔ*L*/*L*_0_ and lg*t* for S1 at different sintering temperature.

**Figure 9.**
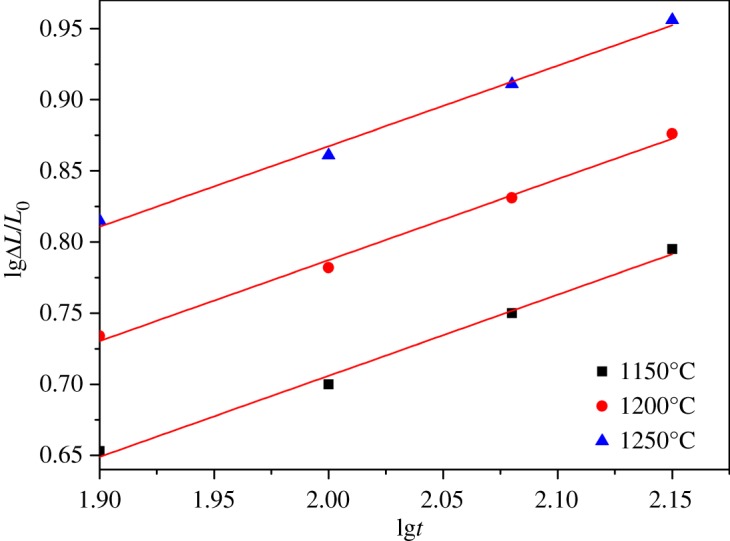
Relation of lgΔ*L*/*L*_0_ and lg*t* for S2 at different sintering temperature.

**Figure 10.**
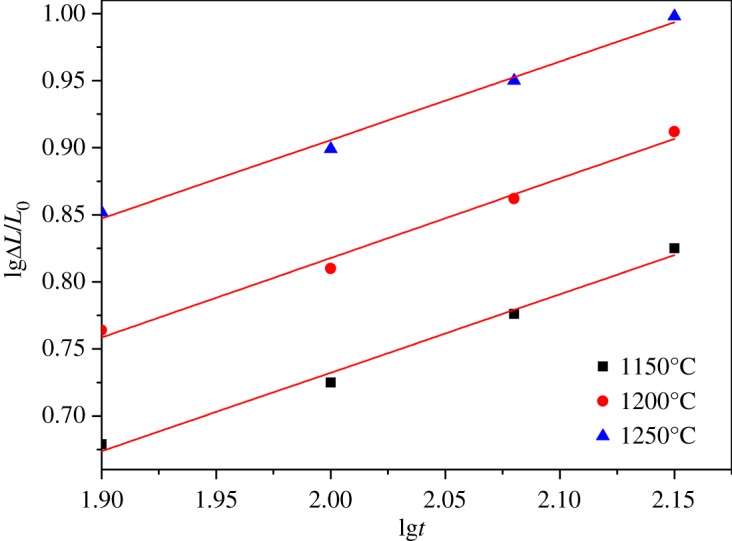
Relation of lgΔ*L*/*L*_0_ and lg*t* for S3 at different sintering temperature.

**Figure 11.**
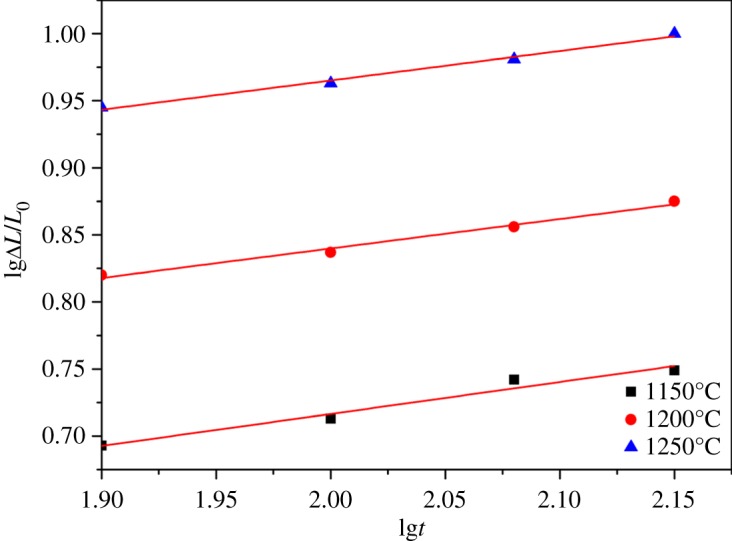
Relation of lgΔ*L*/*L*_0_ and lg*t* for S4 at different sintering temperature.

The linear shrinkage rates of ceramic materials with different sintering temperature and holding time were calculated from equation (2.1) and the results are presented in figures 7–11. It can be seen that linear shrinkage curves exhibit a logarithmic behaviour and a first linear relationship, a straight line is obtained by plotting lgΔ*L*/*L*_0_ versus lg*t*. Based on the above formula, the holding time *t* and the sintering temperature *T* have the following relationship:

3.1 $$\mathrm{lg}t=\mathrm{lg}A^{\prime }+\frac{Q}{R}\cdot \frac{1}{T}.$$

**Figure 12.**
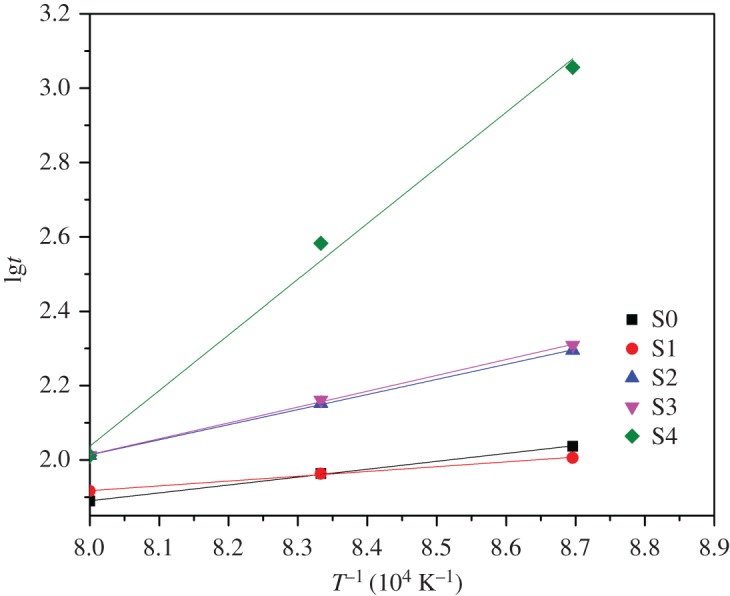
Relation of *T*^−1^ and lg*t* with invariable shrinkage rate.

It can be known that *Q*/*R* is obtained from the slope of a plot of lg*t* versus 1/*T* from the above equation. The liquid-phase sintering apparent activation energy *Q* of ceramic material can be evaluated by using the least-square method from these graphics. When lgΔ*L*/*L*_0_ is a constant value in figures 7–11, the result of lg*t* is shown in table 3, and the relation between 1/*T* and lg*t* with an invariable shrinkage rate is shown in figure 12. As can be seen from the literature [25,26], the densification mechanism of sepiolite nanofibre addition is controlled by a diffusion mechanism.

**Table 3.** Value of lg*t* corresponding to 10^4^ *T*^−1^ with invariable lgΔ*L*/*L*_0_.

sample	10^4^ *T*^−1^			lgΔ*L*/*L*_0_
	8.696	8.333	8.000	
S = 0	2.037	1.963	1.889	0.469
S = 1	2.006	1.963	1.916	0.536
S = 2	2.295	2.152	2.012	0.815
S = 3	2.309	2.161	2.012	0.913
S = 4	3.056	2.583	2.012	0.945

At a certain temperature, the sintering kinetic rate equation can be expressed as follows:

3.2 $$\ln\frac{\Delta L}{L_{0}}=\frac{1}{n}\ln t+\ln A(T),$$

where *n* is the characteristic index of sintering kinetics equation, and *A*(*T*) is a constant associated with interfacial tension, diffusion coefficient and particle radius. Through the establishment of sintering kinetics equation of the ceramic with sepiolite nanofibres, the sintering characteristic index *n* of composite is obtained from the slope of a plot of lnΔ*L*/*L*_0_ versus ln*t*, and the results are shown in table 4. As a result of sintering kinetics studies, the characteristic index of the samples S0 and S1 is from 0.651 to 0.680, the average is 0.66; the index of the samples S2 and S3 is from 1.754 to 1.845, the average is 1.78; the index of the sample S4 is from 4.199 to 4.570, the average is 4.44.

**Table 4.** Characteristic index of ceramic materials toughened by sepiolite nanofibres.

sample	characteristic index			*n* (average)
	1150°C	1200°C	1250°C	
S = 0	0.654	0.651	0.656	0.654
S = 1	0.671	0.668	0.680	0.673
S = 2	1.754	1.757	1.766	1.759
S = 3	1.819	1.765	1.845	1.810
S = 4	4.199	4.551	4.570	4.440

Depending on the literature [27,28], the ceramic sintering process is controlled by volume diffusion when the characteristic index is less than or equal to 2.5. When the sintering process is controlled by grain boundary diffusion, it is greater than or equal to 3. Because of this, we can know that the ceramic sample S2 is controlled by volume diffusion.

## 4. Conclusion

This paper explores the influence of sepiolite nanofibres on the mechanical properties for shellfish porcelain and the sintering kinetics of the reinforced porcelain. Finally, the conclusions drawn from this work are summarized as follows:

(1) With the increase of sepiolite nanofibre addition, the flexural strength and fracture toughness of shellfish porcelain can be improved, but they decreased with greater than a certain amount of nanofibres. The maximum values of the flexural strength and fracture toughness of shellfish porcelain reinforced by 2 wt% sepiolite mineral nanofibres could reach up to 144.5 MPa and 4.5 MPa m^1/2^ from 116.0 MPa and 2.5 MPa m^1/2^, mainly due to crack bridging, fibre pullout and micro-cracks caused by the weak interface between nanofibres and ceramic body.
(2) By plotting lgΔ*L*/*L*_0_ versus lg*t*, using the least-square method to calculate the apparent activation energy *Q* of the ceramic with sepiolite nanofibres, the value is 10.74–124.46 kJ mol^−1^. Thus, we have concluded that the liquid-phase sintering mechanism of ceramic materials containing sepiolite nanofibres is controlled by a diffusion mechanism. The sintering characteristic index *n* of S0, S1, S2 and S3 is less than 2.5, so the migration mechanism in the process of sintering is controlled by the volume diffusion. The sintering characteristic index *n* of S4 is larger than 3, so the migration mechanism in the process of sintering is controlled by the grain boundary diffusion.

## Data accessibility

This article has no additional data.
